# Symptom-Heavy Nutritional Insecurity in Older Adults Initiating Chemotherapy

**DOI:** 10.3390/healthcare14183051

**Published:** 2026-09-17

**Authors:** Jennifer M. Crook, Casey Colin, Eunkyung Lee, Michael Owings, Victoria Loerzel

**Affiliations:** 1 College of Nursing, University of Central Florida, Orlando, FL 32827, USA; michael.owings@ucf.edu (M.O.); victoria.loerzel@ucf.edu (V.L.); 2 Brooks College of Health, University of North Florida, Jacksonville, FL 32224, USA; casey.colin@unf.edu; 3 College of Health Professions & Sciences, University of Central Florida, Orlando, FL 32816, USA; eunkyung.lee@ucf.edu

**Keywords:** nutritional security, cancer nutrition, oncology supportive care, geriatric oncology, patient-reported outcomes, symptom clusters, nutrition vulnerability, social determinants of health

## Abstract

**Highlights:**

**What are the main findings?**

- Nutritional insecurity (NI) is a distinct symptom-heavy phenotype in older adults initiating chemotherapy, marked by consistently higher symptom burden across physical, psychological, and cognitive domains.
- NI was associated with early life interference but not functional decline or inflammatory biomarker abnormalities, indicating a behavioral and psychosocial pathway rather than a biological one.

**What are the implications of the main findings?**

- Symptom clusters may serve as early, clinically actionable indicators of NI, supporting universal NI surveillance in oncology even when traditional access-based screeners or laboratory markers appear normal.
- Findings highlight the need for longitudinal and mechanistic research to clarify NI–symptom–interference pathways and inform targeted interventions, including nutrition services and supportive care.

**Abstract:**

**Background:** Older adults initiating chemotherapy are highly vulnerable to symptom burden and treatment interference. Nutritional insecurity (NI), barriers in food access, preparation, tolerance, and consumption, is a potentially modifiable determinant of treatment vulnerability, yet its role in shaping symptom pathways during chemotherapy is poorly understood. **Objective:** To characterize NI as a symptom phenotype and examine associations between NI and symptom burden, functional limitations, life interference, and inflammatory biomarkers in older adults undergoing chemotherapy. **Methods:** In a cross-sectional analysis nested within a prospective cohort, 79 adults ≥55 years newly diagnosed with cancer and receiving first-line chemotherapy completed NI screening and assessments of physical, psychological, and cognitive symptoms; functional limitations; inflammatory biomarkers (NPAR, NLR, MLR, PLR); and life interference at either pre-chemotherapy (T1) or final chemotherapy (TFinal). Participants were categorized as nutritionally secure (NS) or nutritionally insecure (NI). Group differences were evaluated using chi-square tests, Cramér’s V, Mann–Whitney U tests, ANOVA, and risk differences. **Results:** NI prevalence was 34.2%. Participant demographic and clinical characteristics were largely similar between NI and NS groups in both cohorts. NI participants reported higher prevalence of nearly all symptoms at both time points, with the strongest separation in continuous symptom burden at TFinal (mean rank 33.53 vs. 21.36; *p* = 0.005). Functional limitations showed minimal NI-related differences. Life interference was greater among NI participants in the T1 cohort but not in TFinal. Inflammatory biomarkers did not differ by NI status, and exploratory analyses showed no biomarker-symptom associations. **Conclusions:** NI represents a distinct symptom-heavy phenotype marked by elevated symptom burden and early-life interference, without corresponding functional decline or inflammatory abnormalities. Findings support universal NI surveillance in oncology and motivate longitudinal research to clarify NI–symptom–interference pathways and inform targeted interventions for older adults initiating chemotherapy.

## 1. Introduction

Nutritional security (NS) is a broad and multidimensional concept defined as the biological utilization of accessible, consistent, safe, and nutritious calories, and includes the interplay of food with healthcare, clean water/sanitization, and proper feeding/care practices that support nutrient absorption [1,2]. Despite its relevance, nutritional insecurity (NI) remains poorly differentiated from food insecurity (FI)—barriers to food access [3]—in research and clinical practice. Though FI continues to be widely surveilled and remains an elusive risk factor, the true prevalence of NI is far more difficult to quantify, as individuals may be food secure yet still experience NI [4,5]. Both NI and FI can coexist with being overweight or obese [6,7,8], further complicating risk assessments. Additionally, NI is theorized to contribute to chronic low-grade inflammation and impaired immune response [9,10,11], mechanisms that also underlie fatigue, functional decline, and many other treatment-related symptoms. To date, only a few validated measures exist that capture the full scope of NI [4,5,12,13], and global prevalence estimates remain limited. Recent national data indicate that NI affects 43.9% of US adults overall, but becomes less common with age, with prevalence estimates of 9.9% among adults aged 50–64 and only 5.6% among adults ≥65 years [5].

However, the complexity of NI requires understanding both the contextual factors that impede a nutritionally adequate and high-quality diet intake, as well as the structural constraints that limit a diet’s ability to meet biological nutrient needs [14]. Some older adults may face dietary vulnerabilities from fixed incomes, transportation limitations, cognitive or functional decline, and social isolation [15,16,17]. These factors contribute to NI, linking malnutrition to chronic disease complications, cognitive impairment, and worse outcomes [18,19,20]. Adding a cancer diagnosis and its accompanying treatments may exacerbate nutritional vulnerability in a group already at risk. Chemotherapy intensifies nutrition-related risk by triggering fatigue, gastrointestinal toxicity, immune suppression, and slowed physiologic recovery, sequelae that directly interfere with dietary intake, nutrient absorption, and the ability to maintain adequate nutrition [21]. These treatment-related challenges are already more common in older adults due to multimorbidity and age-related physiological changes, further heightening susceptibility to NI.

Most oncology settings rely on access-based FI measures to identify nutritionally at-risk individuals, which may miss clinically meaningful warning signs [22,23,24] of NI elements that are behavioral, psychosocial, and symptom-driven [25,26,27]. Developing evidence supports that NI is associated with higher symptom burden in older adults [28,29,30]. During chemotherapy, NI may similarly manifest as symptom clusters and functional limitations that signal heightened nutritional vulnerability. Investigating systemic immune-inflammatory biomarkers may also help clarify whether NI corresponds with measurable biological vulnerability during chemotherapy in this population.

Currently, little is known about NI during active cancer treatment [23,24], and no studies have examined NI in older adults undergoing chemotherapy. Therefore, the purpose of this study was to compare symptom prevalence and burden, functional limitations, and life interference between NI and NS older adults undergoing chemotherapy at two clinically relevant time points: pre- and post-chemotherapy. We also aimed to exploratorily examine systemic immune-inflammatory biomarkers, neutrophil-to-percentage ratio (NPAR), neutrophil-lymphocyte ratio (NLR), monocyte-lymphocyte ratio (MLR), and platelet-lymphocyte ratio (PLR) in relation to NI and symptom burden.

## 2. Materials and Methods

### 2.1. Study Design and Participants

This study was a cross-sectional analysis nested within an actively recruiting multi-site prospective cohort of adults aged 50 years undergoing chemotherapy. Participants were recruited from outpatient treatment centers at two cancer centers in Florida. Eligibility criteria for the parent cohort included: age ≥50 years, a recent cancer diagnosis, receipt of chemotherapy on a 2-, 3-, or 4-week cycle, and proficiency in English. Exclusion criteria included prior cancer diagnosis, receipt of chemotherapy before enrollment, and end-stage disease. A recent cancer diagnosis was defined as occurring within the previous 6 months, capturing individuals early in their treatment trajectory and minimizing heterogeneity in pre-treatment symptom burden. We retained these criteria to ensure consistency with the parent study’s design.

For this study, NI assessments were completed at two time points in the parent study to create two distinct cohorts: the pre-chemotherapy visit (T1) or the final chemotherapy visit (TFinal). These time points represent independent cross-sectional samples rather than repeated measures from the same individuals, allowing preliminary characterization of NI-related symptom patterns at two clinically relevant cancer treatment points. For the present cross-sectional analysis, participants were included if they were ≥55 years and completed the NI assessment and the symptom and functional limitation measures at the same visit (T1 or TFinal). Participants were excluded only if NI data were missing. No additional exclusion criteria were applied. Older adult age was defined using the Program of All-Inclusive Care for the Elderly (PACE) criterion, which designates older adulthood beginning at age 55 due to certain medical conditions, including cancer, requiring geriatric-focused care earlier in the disease trajectory [31]. The University of Central Florida Institutional Review Board approved the study (STUDY00003948).

### 2.2. Sample Size Considerations

This study did not employ an a priori sample-size calculation. At the time of study initiation, no published prevalence estimates of nutritional insecurity (NI) among older adults with cancer existed, and therefore no empirically grounded effect-size assumptions were available to support prospective power estimation. The analytical sample for this cross-sectional study thus reflects the number of participants who completed NI screening and symptom assessments at either the pre-chemotherapy (T1) or final chemotherapy (TFinal) visit. Because the study was exploratory, statistical analyses prioritized effect sizes (Cramér’s V, η^2^) and 95% confidence intervals rather than relying solely on *p*-values. No adjustments for multiple comparisons were applied, consistent with the exploratory nature of the work.

### 2.3. Measures

#### 2.3.1. Nutritional Insecurity

NI was assessed using the Nutrition Security Screener (NSS), a validated 4-item screener capturing barriers in access, preparation, tolerance, and consumption (Cronbach’s alpha 0.85) [13]. Validity evidence of the NSS includes robust construct validity supported by factor analysis, moderate correlation with food insecurity (r = 0.63), and strong criterion validity, with higher barrier scores predicting increased odds of diabetes, heart disease, obesity, stroke, and cancer [5]. Classification was based on all NSS items. Participants were categorized as NI if they endorsed “Sometimes”, “Often”, or “Always” on any item, and NS if they endorsed “Never” or “Rarely” across all items.

#### 2.3.2. Symptoms

Symptoms were assessed using the Symptom Representation Questionnaire, a standardized multi-domain checklist capturing physical, psychological, and cognitive symptoms (Cronbach’s alpha 0.63–0.88). Validity evidence includes strong content validity based on the Common-Sense Model, factor-analytic support for symptom-representation domains, and construct validity demonstrated through associations with coping behaviors, help-seeking, and symptom distress [32]. Each symptom was coded dichotomously (present/absent).

#### 2.3.3. Symptom Burden and Composite Scores

A symptom burden score was calculated by summing the number of symptoms reported (range 0–14) into low (0–3), moderate (4–6), and high (7–14) burden categories. Cutoffs were selected to approximate tertile-like groupings while preserving clinically meaningful distinctions between minimal, moderate, and extensive symptom experiences. These categories have been used in prior symptom-cluster research [33,34] and were chosen to balance interpretability with distributional characteristics of the sample. Categorical burden differences were examined using chi-square tests. Continuous burden scores were compared using Mann–Whitney U tests due to non-normal distributions and small sample sizes at TFinal.

#### 2.3.4. Functional Limitations

Functional limitations were assessed by the European Organization for Research and Treatment of Cancer-30 (EORTC-30), which has well-established reliability and validity, supported by decades of psychometric research across cancer types, languages, and countries. Most multi-item scales show acceptable internal consistency (Cronbach’s α ≥ 0.70). Construct validity is supported by factor-analytic confirmation of the scale structure, moderate inter-scale correlations indicating distinct domains, and clear discrimination between patients with differing clinical statuses [35,36]. For this study, participants reported limitations in strenuous activity, long and short walks, need for assistance with ADLs, and limiting effects on work, activity, and leisure. Responses ranged from “Not at All” (1) to “Very Much” (4) and were dichotomized as no limitation (Not at All) versus any limitation (all other responses). NI versus NS differences were evaluated using chi-square tests for the two cohorts at each time point.

#### 2.3.5. Life Interference and Composite Scores

Life interference was assessed using three items asking whether participants’ physical condition or treatment interfered with family life, social activities, or financial stability. Responses ranged from “Not at All” (1) to “Very Much” (4) and were dichotomized as no interference (Not at All) vs. any interference (all other responses). A Life Interference Composite Score (range 0–3) was created by summing the three dichotomized items.

*Interference Composite = Family Interference + Social Interference + Financial Difficulty*

Composite scores were compared using Mann–Whitney U tests. Individual items were examined using chi-square tests and Cramer’s V. A Sankey diagram was constructed to visualize NI to symptom burden to interference pathways.

#### 2.3.6. Inflammatory Biomarkers

Four systemic immune-inflammatory biomarkers were extracted from routine clinical complete blood counts (CBC) with differential values collected within ±3 days of symptom assessment. Biomarkers included neutrophil percentage-to-albumin ratio (NPAR), neutrophil-to-lymphocyte ratio (NLR), monocyte-to-lymphocyte ratio (MLR), and platelet-to-lymphocyte ratio (PLR). These routinely collected hematologic ratios serve as pragmatic indicators of systemic immune-inflammatory activation and have been associated with fatigue, functional decline, and symptom clusters in cancer populations [37,38,39]. All biomarkers were treated as continuous variables and analyzed at three levels:

1. NS vs. NI at each time point. Independent samples ANOVA comparing biomarker means, 95% CIs, *p* values, and effect sizes.
2. Cohort-level T1 vs. TFinal differences. Independent-samples ANOVA comparing biomarker means between NI and NS groups.
3. Exploratory biomarker–symptom analyses. Because inflammatory pathways are frequently implicated in cancer-related symptom burden, we conducted an exploratory analysis to examine whether systemic inflammatory biomarkers were associated with symptom burden of NI/NS participants in each cohort at each time point. Biomarker differences across low, moderate, and high symptom burden categories were analyzed using Kruskal–Wallis tests with Bonferroni-adjusted pairwise comparisons, one-way ANOVA with Levene’s tests, and calculated effect sizes. We also computed Spearman rank order correlations between each biomarker and the composite symptom burden. Inter biomarker correlations were examined to confirm expected physiological coherence.

Evaluating these biomarkers allowed exploratory assessment of whether NI corresponded with measurable immune-inflammatory dysregulation during chemotherapy. Biomarkers were analyzed as continuous variables because no clinically validated categorical cutoffs exist for NPAR, NLR, MLR, or PLR in older adults undergoing chemotherapy.

#### 2.3.7. Statistical Analysis

All analyses were conducted separately for the two independent cohorts in this study: T1 (pre-chemotherapy) and TFinal (post-chemotherapy) using IBM SPSS Statistics, version 30.0. Chi-square tests compared categorical variables (symptoms, functional limitations, interference). Fisher’s Exact Test was not available in the SPSS Base installation used for this study; therefore, analyses with small expected cell counts were interpreted cautiously, and effect sizes (Cramér’s V) were emphasized to contextualize findings. ANOVA compared biomarker means across NI/NS groups and symptom burden categories when assumptions were met. Mann–Whitney U tests evaluated symptoms and interference composites. Missing values were treated as missing completely at random; no imputation was performed. Effect sizes included Cramer’s V for chi-square tests and Eta squared (η^2^) for ANOVA models. Risk differences (%NI—%NS) were calculated for each symptom and functional limitation and visualized using a heat map. Additionally, Sankey diagrams were constructed to illustrate NI-symptom burden-life interference pathways. In all analyses, a two-sided *p* < 0.05 was considered statistically significant.

## 3. Results

### 3.1. Sample Characteristics

A total of 79 unique participants were evaluated in two separate cohorts, with 50 in T1 (assessed at the pre-chemotherapy visit) and 29 in TFinal (assessed at the final chemotherapy visit). Across both cohorts, 34.2% screened positive for NI. Table 1 shows that participant characteristics did not differ significantly between NI and NS groups across race, ethnicity, education, marital status, employment, or cancer stage (all *p* > 0.25) in either cohort. The only cancer-type difference observed was in the TFinal cohort, where GI cancers were more common among NI participants (17.2% vs. 10.3%; *p* = 0.02). Age, albumin, and inflammatory biomarkers were also similar between groups. BMI differed in the post-chemotherapy cohort, where NI participants had higher BMI (32.5 vs. 24.5, *p* = 0.01).

### 3.2. Symptoms Results

Across both time points in each cohort, NI participants reported a higher prevalence of nearly every symptom assessed (Table 2).

#### 3.2.1. Pre Chemotherapy (T1 Cohort)

NI was significantly associated with multiple physical symptoms, including shortness of breath (41.2% vs. 12.2%, V = 0.45), pain (88.2% vs. 39.4%, V = 0.51), and weakness (64.7% vs. 24.2%, V = 0.41). NI participants also reported substantially higher rates of irritability (58.8% vs. 30.3%, V = 0.44) and pain interfering with daily activities, which showed the largest effect size (70.6% vs. 21.2%, V = 0.58). Several additional symptoms, including trouble sleeping, fatigue, appetite loss, and difficulty remembering, showed the same directionality with moderate effect sizes, although these differences were not statistically significant, likely reflecting limited power and small cell counts.

#### 3.2.2. Post-Chemotherapy (TFinal Cohort)

Symptom prevalence was generally higher in the TFinal cohort. The pattern of NI–NS differences remained directionally similar to T1 cohort participants, with NI participants reporting higher rates of shortness of breath (60.0% vs. 21.1%, V = 0.42), weakness (80.0% vs. 47.4%, V = 0.38), fatigue (90.0% vs. 63.2%, V = 0.30), and pain interference (40.0% vs. 15.8%, V = 0.36). However, none of these comparisons reached statistical significance, consistent with smaller sample size and reduced power at TFinal.

### 3.3. Symptom Burden

#### 3.3.1. Pre Chemotherapy (T1 Cohort)

Symptom burden categories differed significantly by NI status (χ^2^ = 23.6, *p* = 0.04, Cramer’s V = 0.69). Over half of NI participants (11 of 17; 64.7%) fell into the high burden category (7–14 symptoms), compared with only 6 of 33 NS participants (18.2%). (Supplemental Table S1) Conversely, low burden (0–3 symptoms) was far more common among NS participants (15 of 33; 45.5%) than NI participants (2 of 17; 11.8%). Mann–Whitney U tests showed higher continuous burden scores among NI participants (mean rank 18.2 vs. 13.3), though this difference did not reach statistical significance (*p* = 0.15) (Supplemental Table S2).

#### 3.3.2. Post-Chemotherapy (TFinal Cohort)

**Figure 1:**
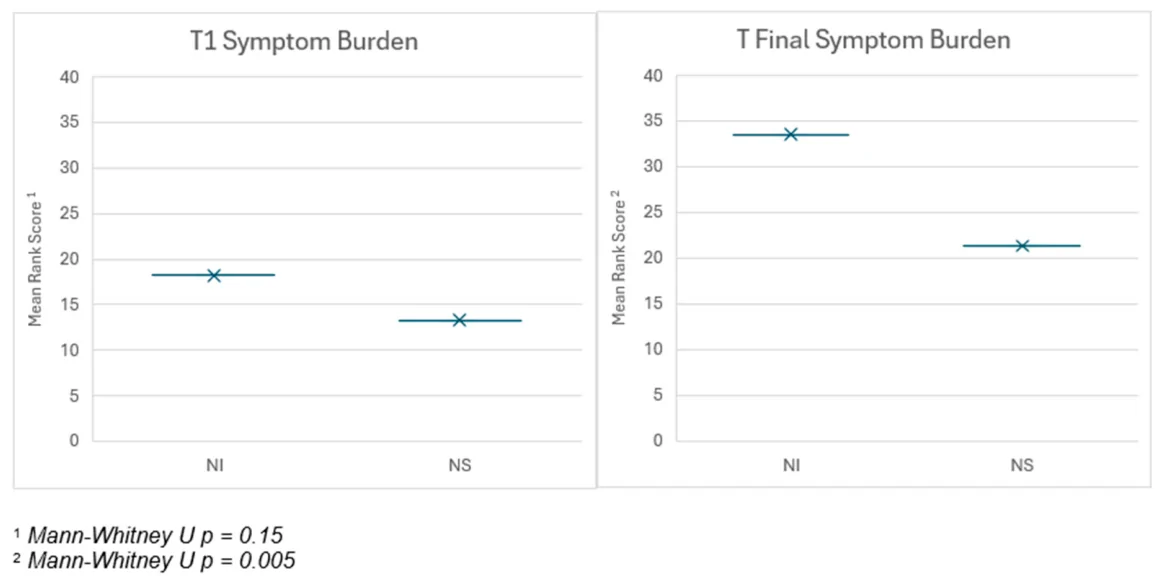
Symptom Burden by Nutritional Insecurity Status in Pre-Chemotherapy (T1) and Final Chemotherapy (TFinal) cohorts.

The distribution of symptom burden showed a similar pattern: NI participants were again more likely to fall into the high burden category (5 of 10; 50%) than NS participants (7 of 19; 36.8%). Due to small, expected cell counts, categorical differences were interpreted descriptively. Continuous burden scores demonstrated a statistically significant difference, with NI participants showing markedly higher symptom burden scores than NS participants (mean rank 33.53 vs. 21.36, *p* = 0.005). This pattern is illustrated in Figure 1.

**Figure 2:**
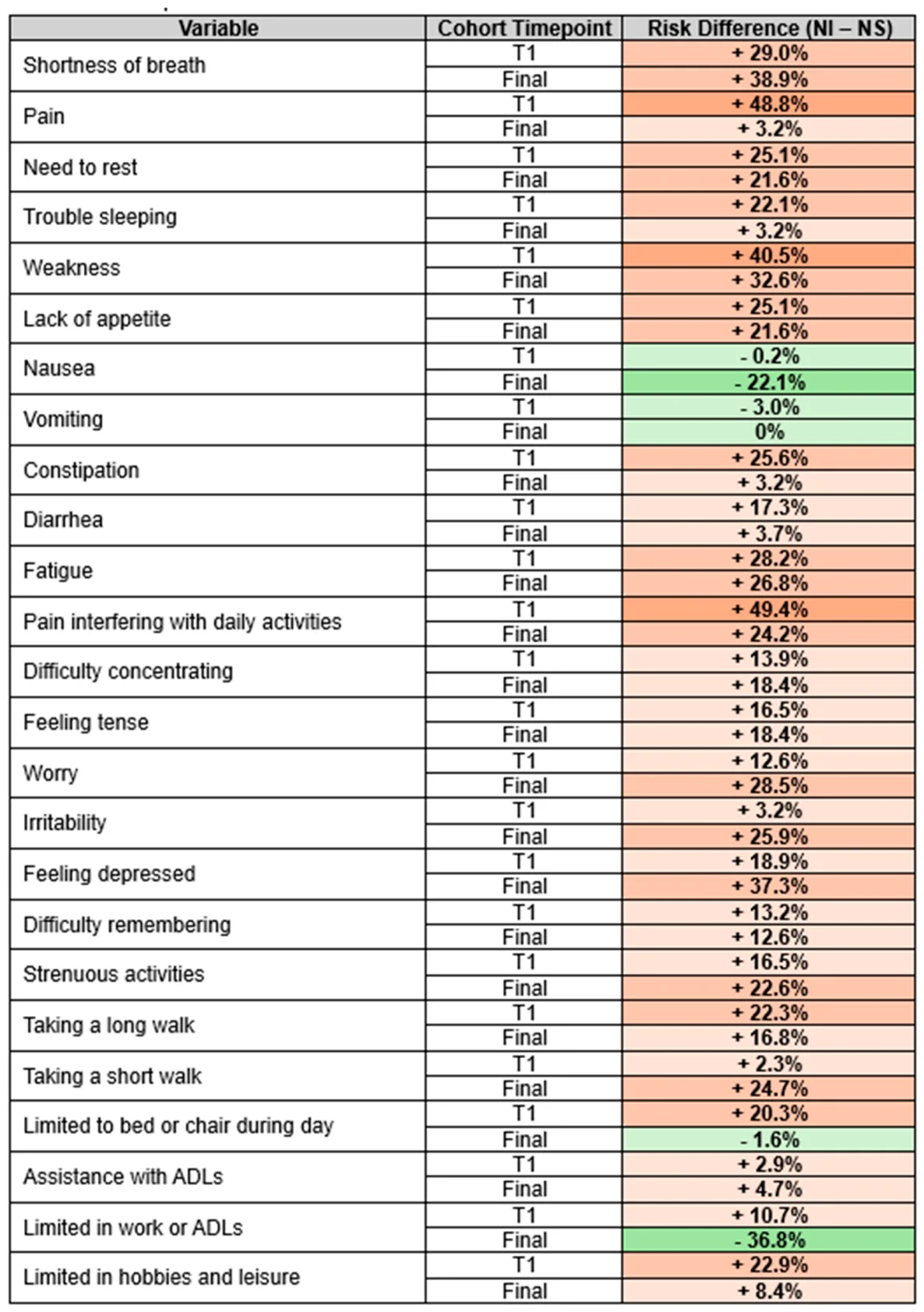
Risk differences (%NI—%NS) in symptom and functional limitation prevalence in pre-chemotherapy (T1) and final chemotherapy (TFinal) cohorts. Positive values indicate higher prevalence among NI participants. Shading reflects higher quartiles of risk difference. Because T1 and TFinal represent independent cross-sectional samples, risk differences are descriptive and should not be interpreted as within-person change.

Risk differences further illustrated the magnitude of NI-associated differences in symptom burden, with the largest contrasts observed for pain (+48.8%), pain interference (+49.4%), weakness (+40.5%), and shortness of breath (+29.0%) in T1 participants. Several additional symptoms, including fatigue, constipation, and appetite loss, also showed meaningful positive differences, whereas nausea and vomiting showed minimal or negative differences. These descriptive contrasts illustrate the substantial symptom load experienced by NI participants (Figure 2).

### 3.4. Functional Limitations Results

Many functional limitations showed generally similar patterns between NI and NS participants at both time points, with most comparisons not reaching statistical significance (Table 3). The only statistically significant difference occurred in the TFinal cohort for short-walk limitation, where NI participants reported substantially higher prevalence than NS participants (30.0% vs. 5.3%; χ^2^ = 6.69, *p* = 0.04, V = 0.48). Across other domains, including strenuous activities, long-walk limitation, bed-or-chair limitation, assistance with ADLs, work/ADL limitation, and hobbies/leisure, NI participants consistently demonstrated higher prevalence than NS participants at both time points, although these differences were descriptive due to small, expected cell counts and not statistically significant.

### 3.5. Life Interference Results

Life interference patterns differed descriptively by NI status in the T1 cohort but not in the TFinal cohort (Table 4). At T1, NI participants were more likely to fall into the high interference category (52.9% vs. 27.3%), although categorical differences were not statistically significant (χ^2^ = 3.35, *p* = 0.19, V = 0.26). Domain-level patterns were directionally similar: NI participants reported higher rates of family interference (47.1% vs. 27.3%), social interference (52.9% vs. 33.3%), and financial difficulty (52.9% vs. 30.3%), with small to moderate effect sizes (V = 0.20–0.22) (Supplemental Tables S2 and S3). Composite interference burden scores showed a borderline difference, with NI participants demonstrating higher mean ranks than NS participants (30.7 vs. 22.8; *p* = 0.057) (Supplemental Table S4; Supplemental Figure S1). The T1 Sankey diagram (Supplemental Figure S3) illustrates these cross-sectional patterns: NI participants were more likely to appear in moderate and high symptom burden categories and, subsequently, in higher interference categories. This relationship between symptom burden and interference was statistically robust at T1 (χ^2^ = 17.9, *p* = 0.001, V = 0.60), as shown in Table 5.

In the TFinal cohort, NI and NS participants reported similar levels of life interference across all composite categories (χ^2^ = 0.04, *p* = 0.98, V = 0.04) and across individual domains (V = 0.02–0.17) (Table 4; Supplemental Table S3). Composite interference burden scores did not differ between groups (mean rank 15.2 vs. 14.1; *p* = 0.76) (Supplemental Table S4; Supplemental Figure S1). The TFinal Sankey diagram (Supplemental Figure S3) similarly shows convergence in interference pathways, with NI and NS participants distributed comparably across symptom burden and interference categories. As in the T1 cohort, interference categories were strongly associated with symptom burden (χ^2^ = 17.0, *p* = 0.001, V = 0.80; Table 5), but without NI-specific differences.

#### 3.5.1. Inflammatory Biomarkers Results

##### NI vs. NS in T1 and TFinal Cohorts

Across all biomarkers (NPAR, NLR, MLR, PLR), no NI/NS differences were statistically significant in both cohorts at either time point (Supplemental Tables S5 and S6). Mean values and confidence intervals overlapped substantially, F-statistics were small (F = 0.01–1.22), and effect sizes were trivial (η^2^ = 0.00–0.05) (Supplemental Tables S5 and S6).

##### Cohort-Level Differences (T1 vs. TFinal)

Only MLR differed between cohorts, found to be significantly higher in the TFinal cohort (F = 5.04, *p* = 0.03, η^2^ = 0.09) (Supplemental Table S7). NPAR, NLR, and PLR did not differ between cohorts (all *p* > 0.28).

##### Exploratory Biomarker-Symptom Analyses

Exploratory analyses showed no evidence that systemic inflammatory biomarkers were associated with symptom burden in either cohort at either time point. Biomarker levels did not differ across low, moderate, or high symptom burden categories (all *p* > 0.32), and Spearman correlations between each biomarker and composite symptom burden were uniformly weak and nonsignificant (ρ = −0.16–0.17; Supplemental Table S8). In contrast, biomarkers were strongly intercorrelated (ρ = 0.53–0.92; all *p* < 0.01; Supplemental Table S9), reflecting expected physiological coherence among inflammatory ratios but no measurable relationship with symptom burden or NI status.

## 4. Discussion

This cross-sectional analysis identified nutritional insecurity (NI) as a ubiquitous (34.2%), symptom-heavy phenotype among older adults undergoing chemotherapy. These findings conflict with early evidence that NI decreased with age in US adults [5], highlighting the necessity of nutritional surveillance in oncology settings where treatments may introduce or exacerbate the contextual factors inherent in NI. Participant characteristics in this study were broadly similar between NI and NS groups, suggesting that NI did not operate as a demographic or clinical confounder in this sample, strengthening the interpretability of downstream comparisons in symptoms, functional limitations, and life interference. Although NI is often overlooked in individuals without traditional food-insecurity risk profiles, the pattern observed here, particularly the higher BMI among NI participants, is consistent with evidence that NI can coexist with overweight and obesity [40,41,42].

Across two independent cohorts, NI was consistently associated with higher prevalence of physical, psychological, and cognitive symptoms, and with greater early life interference. These findings align with emerging evidence that NI reflects behavioral and psychosocial vulnerability rather than solely access-based food insecurity [13], and that symptom clusters may serve as clinically meaningful indicators of nutritional risk in older adults [43,44]. The biggest NI-related differences were observed in symptom burden. At both time points, NI participants in each cohort reported higher prevalence of nearly all symptoms assessed, with particularly large contrasts in pain, pain interference, weakness, shortness of breath, and fatigue. Continuous symptom burden showed the clearest separation at the final chemotherapy visit (TFinal cohort), where NI participants demonstrated substantially higher mean ranks. These patterns suggest that NI may correspond with heightened symptom experience during chemotherapy, even when demographic and clinical characteristics are similar. Because the cohorts represent independent samples, these findings reflect cross-sectional differences rather than within-person change.

Life interference also differed by NI status, but only at the pre-chemotherapy visit. NI participants reported higher rates of family, social, and financial interference, and composite interference scores reflecting the broader life impacts associated with NI [45,46,47]. Sankey diagrams illustrated that NI participants were more likely to appear in moderate and high symptom burden categories and subsequently in higher interference categories. These descriptive pathways were supported by strong associations between symptom burden and interference at both time points. In the final chemotherapy cohort, however, NI and NS participants reported similar interference levels, suggesting that early interference may be more sensitive to NI-related vulnerabilities than later treatment experiences.

In contrast, functional limitations showed minimal NI-related differences. Although NI participants reported higher prevalence of several limitations, most comparisons were nonsignificant, and effect sizes were small. Only short-walk limitations in the TFinal cohort reached statistical significance, and several comparisons had small, expected cell counts, warranting cautious interpretation. These findings suggest that NI may influence subjective symptom experience and life interference more strongly than functional status during chemotherapy. This pattern may reflect the lived reality of NI in that individuals may normalize chronic strain or maintain daily responsibilities despite symptom burden because work must be done, bills must be paid, and caregiving cannot pause [43,44]. Such contextual pressures may blunt the observable impact of cancer-related symptoms on daily functioning, reflecting the universalizing effect of chemotherapy: as treatment progresses, symptom-related interference becomes widespread, reducing the relative impact of NI-specific strain.

Inflammatory biomarkers (NPAR, NLR, MLR, PLR) did not differ between NI and NS participants in either cohort at either time point. Exploratory analyses showed no biomarker differences across symptom burden categories and no meaningful correlations between biomarkers and symptom burden, contradicting findings from non-cancer populations where FI and poor diet quality are linked to inflammatory regulation [45]. Biomarkers were strongly correlated, reflecting expected physiological coherence, but showed no relationship with NI or symptom burden. These findings suggest that NI-related symptom phenotype patterns may be driven more by behavioral, psychosocial, or contextual mechanisms than by measurable systemic immune-inflammation in this sample.

Several contextual factors may help explain these findings. Cultural influence on food access, dietary practices, and symptom interpretation shape participants’ nutritional experiences. The sample included individuals identifying as Black/African American, White, Asian, and multiracial, each of whom may face distinct cultural norms, caregiving structures, and dietary preferences that influence NI. These cultural dimensions were not directly measured in this study, but warrant explicit consideration in future research, particularly given known disparities in food access, caregiving support, and symptom reporting across cultural groups [5]. Age-related vulnerabilities, including fixed incomes, functional decline, and cognitive changes, may shape nutritional experiences during chemotherapy. Cancer treatment-related symptoms such as fatigue, gastrointestinal toxicity, appetite changes, and cognitive strain may also interact with NI-related barriers in food access, preparation, and tolerance, potentially amplifying nutritional vulnerability during treatment.

This study has several limitations. The cross-sectional study design precludes causal inference and prevents determination of temporality between NI and symptom burden. The analytical sample was derived from a parent cohort with different primary objectives, introducing potential selection bias. Several variables had missing values, and small expected cell counts limited the interpretability of some chi-square tests. The absence of a formal sample-size calculation and the modest sample size reduce statistical precision, particularly for subgroup analyses and less prevalent symptoms. Analyses were exploratory and did not adjust for multiple comparisons; findings should therefore be interpreted cautiously. In the future, larger study groups must be examined to validate findings reported here. Sample estimates of future efforts need to utilize approximately 52 participants (26 per group) to achieve 80% power at α = 0.05. Living arrangement and comorbidity data, both relevant to nutritional vulnerability, were not collected in either cohort. Comorbidities such as diabetes, dysphagia, kidney disease, and inflammatory bowel conditions, which may influence NI and dietary behaviors, were neither collected nor explored. Treatment-specific factors (e.g., regimen intensity, cycle length, toxicity profiles) that may interact with NI and influence symptom burden were not assessed. Additionally, cancer-type heterogeneity, particularly gastrointestinal malignancies that directly affect appetite, digestion, and nutrient absorption, may contribute to variation in NI-related symptom patterns. The sample included diverse cancer types, and although GI cancers were more common among NI participants at TFinal, the study was not powered to examine cancer-type or age-stratified effects.

Despite these limitations, the study offers several strengths. It is among the first to characterize NI in older adults undergoing chemotherapy, integrating symptom burden, functional limitations, life interference, and inflammatory biomarkers. The multidimensional assessment provides a comprehensive view of NI-related vulnerability and effect sizes complement *p*-values to contextualize findings. The use of Sankey diagrams offers a novel visualization of NI-symptom-interference pathways.

These findings have several important implications for oncology practice. Symptom clusters may be an early clinical sign of NI, where functional limitations, however, may not be reliable indicators of NI in older adults undergoing chemotherapy. Clinicians should not wait for functional decline to intervene. Life interference may be highest at treatment initiation, suggesting that early supportive care, social work engagement, and medical nutrition therapy from a registered dietitian may be most impactful. Early referral to a registered dietitian for medical nutrition therapy interventions for those with any cancer diagnosis is recommended, though current referral processes are frequently delayed or not initiated at all in this population [4,46,47]. Interestingly, BMI is not protective and may obscure NI risk; clinicians should avoid relying on weight status to infer nutritional inadequacy. By waiting until weight loss has occurred to refer the patient to nutrition interventions, care becomes focused on damage control rather than preserving and building nutrient stores [48].

Overall, NI appears to represent a distinct symptom-heavy phenotype marked by elevated symptom burden and early-life interference, without corresponding functional decline or inflammatory abnormalities. These findings support universal NI surveillance in oncology, even when traditional access-based screeners or laboratory markers appear normal. Future longitudinal studies with larger samples are needed to clarify NI-symptom-interference pathways, examine cancer-type heterogeneity, incorporate cultural and comorbidity data, and evaluate whether targeted nutrition interventions can mitigate symptom burden and improve treatment experiences for older adults undergoing chemotherapy.

## 5. Conclusions

Nutritional insecurity (NI) emerged as a distinct, symptom-heavy phenotype among older adults undergoing chemotherapy. Across two independent cohorts at treatment time points, NI was consistently associated with higher prevalence of physical, psychological, and cognitive symptoms, and with greater early life interference, even though demographic and clinical characteristics, including cancer type, stage, and inflammatory biomarkers, were largely similar between NI and nutritionally secure participants. These findings suggest that NI reflects behavioral and psychosocial vulnerability that may not be apparent through traditional access-based screeners, laboratory markers, or demographic profiles.

Functional limitations and systemic inflammatory biomarkers did not differ meaningfully by NI status, indicating that NI-related vulnerability may manifest primarily through subjective symptom experience and early interference rather than measurable functional decline or inflammatory dysregulation. This pattern underscores the importance of incorporating symptom-based indicators into nutritional risk surveillance, particularly for older adults whose nutritional challenges may be driven by treatment-related symptoms, cultural dietary practices, caregiving structures, or other contextual factors not captured by conventional food-access measures.

Given the cross-sectional design, these findings represent associations rather than causal pathways. Larger, longitudinal studies are needed to clarify temporal relationships between NI, symptom burden, and life interference; examine cancer-type heterogeneity; incorporate comorbidity and living-arrangement data; and evaluate whether targeted nutrition interventions can mitigate symptom burden and improve treatment experiences. Collectively, this study supports universal NI surveillance in oncology and highlights the need for mechanistic and longitudinal research to better understand NI-related symptom pathways and inform clinically actionable strategies for older adults initiating chemotherapy.

## Figures and Tables

**Table 1:** Participant Characteristics by Nutritional Insecurity Status and Time Point (T1 and TFinal cohorts).

Characteristic		T1 NS ^a^	T1 NI ^a^	T1 ^b^ Test Statistic	T1 *p*	T Final NS ^a^	T Final NI ^a^	TFinal ^b^ Test Statistic	TFinal *p*
**Sex**	Male	6 (18.2%)	3 (17.6%)	1.46	0.48	5 (26.3%)	3 (30%)	0.05	0.83
	Female	13 (39.4%)	4 (23.5%)			14 (73.7%)	7 (70%)		
	Missing	14 (42.4%)	10 (58.8%)			0 (0%)	0 (0%)		
**Race**	White	21 (63.6%)	11 (64.7%)	6.49	0.26	11 (57.9%)	7 (70%)	3.43	0.33
	Black/African American	7 (21.2%)	4 (23.5%)			3 (15.8%)	3 (30%)		
	Asian	2 (6.1%)	0 (0%)			4 (21.1%)	0 (0%)		
	Other/Multi	3 (9.1%)	1 (5.9%)			1 (5.2%)	0 (0%)		
	Missing	0 (0%)	1 (5.9%)			0 (0%)	0 (0%)		
**Hispanic** **ethnicity**	Yes	6 (18.2%)	2 (11.8%)	0.54	0.77	4 (21.1%)	5 (50%)	2.57	0.11
	No	26 (78.8%)	14 (82.4%)			15 (78.9%)	5 (50%)		
	Missing	1 (3%)	1 (5.9%)			0 (0%)	0 (0%)		
**Education**	High school or less	6 (18.2%)	4 (23.5%)	2.78	0.60	3 (15.8%)	1 (10%)	4.95	0.18
	Some college/trade	11 (33.3%)	6 (35.3%)			2 (10.5%)	4 (40%)		
	College degree	16 (48.5%)	6 (35.3%)			14 (73.7%)	5 (50%)		
	Missing	0 (0%)	1 (5.9%)			0 (0%)	0 (0%)		
**Marital Status**	Married/partnered	18 (54.5%)	6 (35.3%)	2.12	0.83	12 (63.2%)	6 (60%)	2.44	0.49
	Divorced/separated	8 (24.2%)	6 (35.3%)			4 (21.1%)	4 (40%)		
	Never married	4 (12.1%)	3 (17.6%)			1 (5.3%)	0 (0%)		
	Widowed	2 (6.1%)	1 (5.9%)			2 (10.5%)	0 (0%)		
	Missing	1 (3.0%)	1 (5.9%)			0 (0%)	0 (0%)		
**Support at Home ^c^**	None	0 (0%)	2 (11.8%)	5.01	0.17				
	At least one person	33 (100%)	15 (88.2%)						
**Employment**	Retired	15 (45.5%)	5 (29.4%)	5.12	0.28	8 (42.1%)	4 (40%)	0.60	0.74
	Working (FT/PT)	15 (45.5%)	10 (58.8%)			10 (52.6%)	6 (60%)		
	Disabled	3 (9%)	1 (5.9%)			1 (5.3%)	0 (0%)		
	Missing	0 (0%)	1 (5.9%)			0 (0%)	0 (0%)		
**Cancer Type**	Breast	4 (12.1%)	2 (11.8%)	0.001	0.97	4 (21.1%)	1 (10.0%)	0.56	0.45
	GI (colon, pancreas, rectal, esophageal, liver)	6 (18.2%)	1 (5.9%)	4.64	0.20	3 (15.8%)	5 (50%)	5.54	**0.02**
	GU (prostate, bladder, renal)	4 (12.1%)	0 (0%)	1.07	0.30	1 (5.2%)	0 (0%)	0.55	0.46
	Gynecologic (endometrial, ovarian, uterine, fallopian)	3 (9.0%)	1 (5.9%)	0.53	0.47	4 (21.1%)	2 (20%)	0.23	0.63
	Lung	2 (6.1%)	0 (0%)	1.07	0.30	2 (10.5%)	1 (10%)	0.002	0.97
	Hematologic/Skin	6 (18.2%)	3 (17.6%)	3.26	0.07	5 (26.3%)	1 (10%)	0.19	0.67
	Missing	6 (18.2%)	1 (5.9%)			0 (0%)	0 (0%)		
**Cancer Stage**	I-II	2 (6.1%)	3 (17.7%)	8.04	0.24	3 (15.8%)	1 (10%)	0.91	0.92
	III-IV	9 (27.3%)	4 (23.5%)			11 (57.9%)	7 (70%)		
	Unable to stage or Unstaged	7 (21.2%)	0 (0%)			5 (26.3%)	2 (20%)		
	Missing	15 (45.5%)	10 (58.8%)			0 (0%)	0 (0%)		
**Age in years**		63.8 ± 7.1	62.2 ± 7.1	0.55	0.46	64 ± 9.2	64.6 ± 6.6	0.03	0.86
**BMI ^d^**		28.5 ± 10	33.0 ± 5.2	1.28	0.27	24.5 ± 4.3	32.5 ± 7.7	8.46	**0.01**
**Albumin**		3.87 ± 0.48	4.04 ± 0.38	0.71	0.41	4.01 ± 0.35	3.68 ± 0.38	4.44	0.06
**NPAR ^e^**		16.0 ± 4.1	14.4 ± 6.5	0.58	0.46	15.2 ± 5.5	15.0 ± 3.4	0.01	0.92
**NLR ^f^**		3.0 ± 2.4	2.1 ± 1.5	0.86	0.36	4.90 ± 7.2	2.70 ± 2.4	0.78	0.39
**MLR ^g^**		0.37 ± 0.2	0.32 ± 0.2	0.30	0.59	0.54 ± 0.3	0.62 ± 0.6	0.23	0.64
**PLR ^h^**		177 ± 111	128 ± 59.7	1.22	0.28	206 ± 253	142 ± 68.2	0.55	0.47

**^a^:** categorical variables presented as *n* (%), continuous variables presented as mean(SD); **^b^:** χ^2^ for categorical variables; F for continuous variables; **^c^:** not assessed in TFinal cohort; **^d^:** Body Mass Index (kg/m^2^); **^e^:** neutrophil percentage to albumin ratio; **^f^:** neutrophil to lymphocyte ratio; **^g^:** monocyte to lymphocyte ratio; **^h^:** platelet to lymphocyte ratio.

**Table 2:** Symptom Prevalence by NI Status in Pre-Chemotherapy (T1) and Final Chemotherapy (TFinal) cohorts.

Symptom	Cohort Time Point	NS *n* (%)	NI *n* (%)	χ^2^	*p*	V
**Shortness of breath**	T1	4 (12.2)	7 (41.2)	10.2	**0.02**	**0.45**
	Final	4 (21.1)	6 (60.0)	5.22	0.16	0.42
**Pain**	T1	13 (39.4)	15 (88.2)	12.8	**0.005**	**0.51**
	Final	7 (36.8)	4 (40.0)	3.71	0.30	0.36
Need to rest	T1	15 (45.5)	12 (70.6)	2.91	0.41	0.24
	Final	13 (68.4)	9 (90.0)	4.55	0.21	0.40
Trouble sleeping	T1	16 (48.5)	12 (70.6)	6.49	0.09	0.36
	Final	7 (36.8)	4 (40.0)	2.07	0.56	0.27
**Weakness**	T1	8 (24.2)	11 (64.7)	8.49	**0.04**	**0.41**
	Final	9 (47.4)	8 (80.0)	4.29	0.23	0.38
Lack of appetite	T1	9 (27.3)	8 (47.1)	6.13	0.11	0.35
	Final	6 (31.6)	4 (40.0)	1.98	0.37	0.26
Nausea	T1	2 (6.1)	1 (5.9)	2.97	0.23	0.24
	Final	8 (42.1)	2 (20.0)	1.61	0.45	0.24
Vomiting	T1	1 (3.0)	0 (0)	0.53	0.47	0.10
	Final	0 (0)	0 (0)	.	.	.
Constipation	T1	9 (27.3)	9 (52.9)	5.01	0.17	0.32
	Final	7 (36.8)	4 (40.0)	0.23	0.97	0.09
Diarrhea	T1	4 (12.1)	5 (29.4)	3.26	0.20	0.26
	Final	5 (26.3)	3 (30.0)	2.44	0.49	0.29
Fatigue	T1	14 (42.4)	12 (70.6)	6.51	0.09	0.36
	Final	12 (63.2)	9 (90.0)	2.53	0.47	0.30
**Pain in daily activities**	T1	7 (21.2)	12 (70.6)	16.5	**0.002**	**0.58**
	Final	3 (15.8)	4 (40.0)	3.78	0.15	0.36
Difficulty concentrating	T1	9 (27.3)	7 (41.2)	1.09	0.78	0.15
	Final	6 (31.6)	5 (50.0)	2.39	0.30	0.29
Feeling tense	T1	12 (36.4)	9 (52.9)	2.50	0.48	0.22
	Final	6 (31.6)	5 (50.0)	2.39	0.30	0.29
Worry	T1	20 (60.6)	12 (70.6)	3.36	0.34	0.26
	Final	9 (47.4)	6 (60.0)	2.63	0.45	0.30
**Irritability**	T1	10 (30.3)	10 (58.8)	9.75	**0.02**	**0.44**
	Final	7 (36.8)	4 (40.0)	4.41	0.11	0.39
Feeling depressed	T1	7 (21.2)	8 (47.1)	7.33	0.12	0.38
	Final	4 (21.1)	4 (40.0)	1.18	0.56	0.20
Difficulty remembering	T1	11 (33.3)	12 (70.6)	7.56	0.06	0.39
	Final	7 (36.8)	5 (50.0)	2.31	0.51	0.28

**Table 3:** Functional Limitations by NI Status in Pre-Chemotherapy (T1) and Final Chemotherapy (TFinal) Cohorts.

Functional Limitation	Cohort Time Point	NS *n* (%)	NI *n* (%)	χ^2^	*p*	V
Strenuous activities	T1	12 (36.4)	9 (52.9)	3.02	0.56	0.25
	Final	9 (47.4)	7 (70.0)	2.31	0.51	0.28
Taking a long walk	T1	14 (42.4)	11 (64.7)	2.79	0.43	0.24
	Final	12 (63.2)	8 (80.0)	4.02	0.26	0.37
**Taking a short walk**	T1	7 (21.2)	4 (23.5)	2.47	0.48	0.22
	Final	1 (5.3)	3 (30.0)	6.69	**0.04**	**0.48**
Limited to bed or chair during day	T1	3 (9.1)	5 (29.4)	6.01	0.11	0.35
	Final	6 (31.6)	3 (30.0)	2.22	0.33	0.28
Assistance with ADLs	T1	1 (3.0)	1 (5.9)	0.24	0.63	0.07
	Final	1 (5.3)	1 (10.0)	0.23	0.63	0.09
Limited in work or ADLs	T1	12 (36.4)	8 (47.1)	0.65	0.89	0.11
	Final	7 (76.8)	4 (40.0)	3.18	0.37	0.33
Limited in hobbies and leisure	T1	8 (24.2)	8 (47.1)	3.98	0.26	0.28
	Final	6 (31.6)	4 (40.0)	0.35	0.95	0.11

**Table 4:** Life Interference Burden Composite Categories by NI Status and Time Point in T1 and TFinal Cohorts.

T1 Cohort					
Life Interference Burden Category	NS *n* (%)	NI *n* (%)	χ^2^	*p*	V
Low (0)	14 (42.4)	4 (23.5)	3.35	0.19	0.26
Moderate (1)	10 (30.3)	4 (23.5)			
High (2–3)	9 (27.3)	9 (52.9)			
**T Final Cohort**					
**Life Interference Burden** **Category**	**NS** ***n*** **(%)**	**NI** ***n*** **(%)**	**χ^2^**	***p***	**V**
Low (0)	6 (33.3)	3 (30.0)	0.04	0.98	0.04
Moderate (1)	7 (38.9)	4 (40.0)			
High (2–3)	5 (27.8)	3 (30.0)			

**Table 5:** Symptom burden categories (low, moderate, high) by life interference burden (0–3) in pre-chemotherapy (T1) and final chemotherapy (TFinal) cohorts.

T1 Cohort						
Interference Category (0–3)	Low Symptom Burden	Moderate Symptom Burden	High Symptom Burden	χ^2^	*p*	V
Life Interference Low (0)	11 (22.0)	6 (12.0)	1 (2.0)	17.9	**0.001**	**0.60**
Life Interference Moderate (1)	4 (8.0)	6 (12.0)	4 (8.0)			
Life Interference High (2–3)	2 (4.0)	4 (8.0)	12 (24.0)			
**T Final Cohort**						
**Interference Category** **(0–3)**	**Low** **Symptom Burden**	**Moderate Symptom Burden**	**High Symptom Burden**	**χ^2^**	***p***	**V**
Life Interference Low (0)	5 (17.9)	2 (7.1)	2 (7.1)	18.0	**0.001**	**0.80**
Life Interference Moderate (1)	0 (0)	8 (28.6)	3 (10.7)			
Life Interference High (2–3)	0 (0)	2 (7.1)	6 (21.4)			

**Note:** One participant in the TFinal cohort did not complete the life interference items; therefore, interference categories sum to 28 rather than 29.

## Data Availability

The parent study from which this cross-sectional analysis is drawn is still actively recruiting and collecting data. Because the full dataset is not yet complete and ongoing data collection could be affected by premature public release, access is limited to researchers with a justified need for the data and will be provided upon request.

## Supplementary Materials

The following supporting information can be downloaded at: https://www.mdpi.com/article/10.3390/healthcare14183051/s1, Figure S1. NI/NS Life Interference Composite Burden by NI Status in Pre-Chemotherapy (T1) and Final Chemotherapy (TFinal) Cohorts; Figure S2. Sankey Diagram of NI, Symptom Burden, and Life Interference Pathways in Pre-Chemotherapy (T1) Cohort; Figure S3. Sankey Diagram of NI, Symptom Burden, and Life Interference Pathways in Final Chemotherapy (TFinal) Cohort. Table S1. Symptom Burden Categories by NI Status in Pre-Chemotherapy (T1) and Final Chemotherapy (TFinal) Cohorts; Table S2. Mann Whitney U Tests for Continuous Symptom Burden in Pre-Chemotherapy (T1) and Final Chemotherapy (TFinal) Cohorts; Table S3. Life Interference Burden by Timepoint in T1 and TFinal Cohorts; Table S4. Mann-Whitney U test of Life Interference Composite Burden by NI/NS Status and Timepoint in T1 and TFinal Cohorts; Table S5. Biomarker Differences Between NS and NI Participants in Pre-Chemotherapy (T1) Cohorts; Table S6. Biomarker Differences Between NS and NI Participants in Final Chemotherapy (TFinal) Cohort; Table S7. Cohort-Level Biomarker Differences Between Pre-Chemotherapy (T1) and Final Chemotherapy (TFinal) Cohorts; Table S8. Spearman Correlations Between Biomarkers and Symptom Burden in Pre-Chemotherapy (T1) and Final Chemotherapy (TFinal) Cohorts; Table S9. Spearman Intercorrelations Among Immune-Inflammatory Biomarkers (NPAR, NLR, MLR, PLR).

## Abbreviations

The following abbreviations are used in this manuscript:

NI	Nutritional Insecurity
NPAR	Neutrophil Percentage-to-Albumin Ratio
NLR	Neutrophil-to-Lymphocyte Ratio
MLR	Monocyte-to-Lymphocyte Ratio
PLR	Platelet-to-Lymphocyte Ratio
NS	Nutritional Security
FI	Food Insecurity
ANOVA	Analysis of Variance
NSS	Nutrition Security Screener
EORTC-30	European Organization for Research and Treatment of Cancer-30
CBC	Complete Blood Count
