# Supplementary material for: Symptom-Heavy Nutritional Insecurity in Older Adults Initiating Chemotherapy
Source: Healthcare (Basel). 2026 Sep 17;14(18):3051. doi: 10.3390/healthcare14183051 (PMC13606286; doi:10.3390/healthcare14183051)
Supplement: Supplementary file 1 [file healthcare-14-03051-s001.zip › healthcare-4481282-supplementary.pdf]

Supplemental Table S1. Symptom Burden Categories by NI Status in Pre-Chemotherapy (T1) and Final Chemotherapy (TFinal) Cohorts

T1 Cohort

Symptom Burden Category	NS	NI	χ^2	*p*	V
Low (0-3)	15	2	23.6	**0.04**	**0.69**
Moderate (4-6)	12	4			
High (7-14)	6	11			

T Final Cohort

Symptom Burden Category	NS	NI	χ^2	*p*	V
Low (0-3)	4	1	9.60	0.57	0.41
Moderate (4-6)	8	4			
High (7-14)	7	5			

Note: χ^2 values for TFinal should be interpreted cautiously due to small expected cell counts; Cramer's V is provided for descriptive effect size.

Supplementary Table S2. Mann-Whitney U Tests for Continuous Symptom Burden in Pre-Chemotherapy (T1) and Final Chemotherapy (TFinal) Cohorts

T1 Cohort

Group	Mean Rank	U	SE	*p*
NI	18.2	127	21.7	0.15
NS	13.3			

T Final Cohort

Group	Mean Rank	U	SE	*p*
NI	33.53	417	48.6	**0.005**
NS	21.36			

Supplemental Table S3. Life Interference Burden by Timepoint in T1 and TFinal Cohorts

Life Interference Domain	Cohort Timepoint	NS n (%)	NI n (%)	χ^2	*p*	V
Family	T1	9 (27.3)	8 (47.1)	1.96	0.16	0.20
	Final	3 (15.8)	3 (30.0)	0.81	0.37	0.17
Social	T1	11 (33.3)	9 (52.9)	8.41	0.34	0.20
	Final	8 (42.1)	4 (40.0)	0.01	0.91	0.02
Financial	T1	10 (30.3)	9 (52.9)	2.44	0.12	0.22
	Final	8 (44.4)	5 (50.0	0.08	0.78	0.05

Supplemental Table S4. Mann-Whitney U test of Life Interference Composite Burden by NI/NS Status and Timepoint in T1 and TFinal Cohorts

T1 Cohort

Group	Mean Rank	U	SE	*p*
NI	30.7	369.5	46.7	0.057
NS	22.8			

T Final Cohort

Group	Mean Rank	U	SE	*p*
NI	15.2	97.0	19.8	0.76
NS	14.1			

Supplemental Figure S1. NI/NS Life Interference Composite Burden by NI Status in Pre-Chemotherapy (T1) and Final Chemotherapy (TFinal) Cohorts

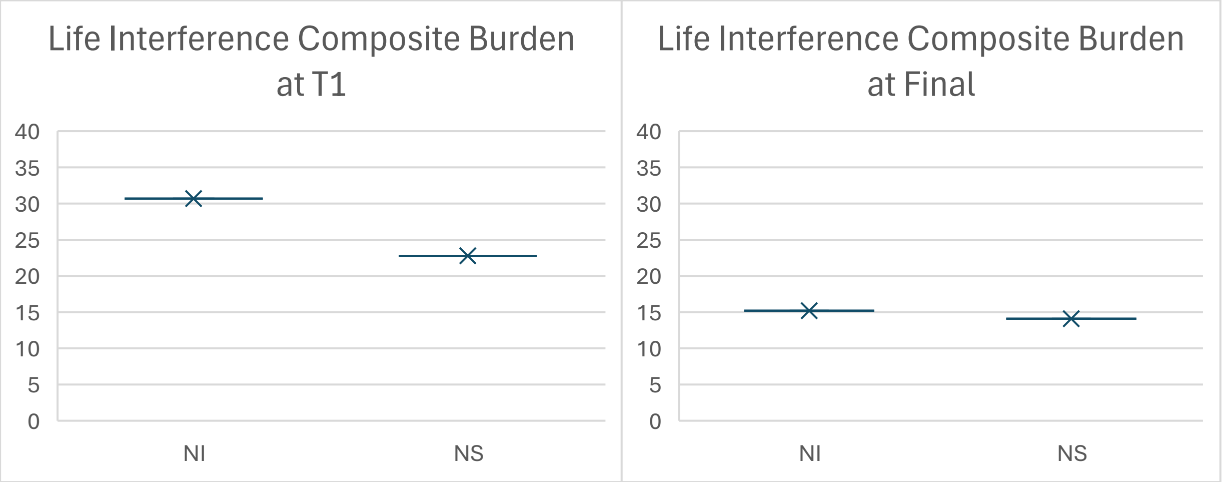

[1] *Mann-Whitney U p = 0.057*
[2] *Mann-Whitney U p = 0.76*

Supplemental Figure S2. Sankey Diagram of NI, Symptom Burden, and Life Interference Pathways in Pre-Chemotherapy (T1) Cohort

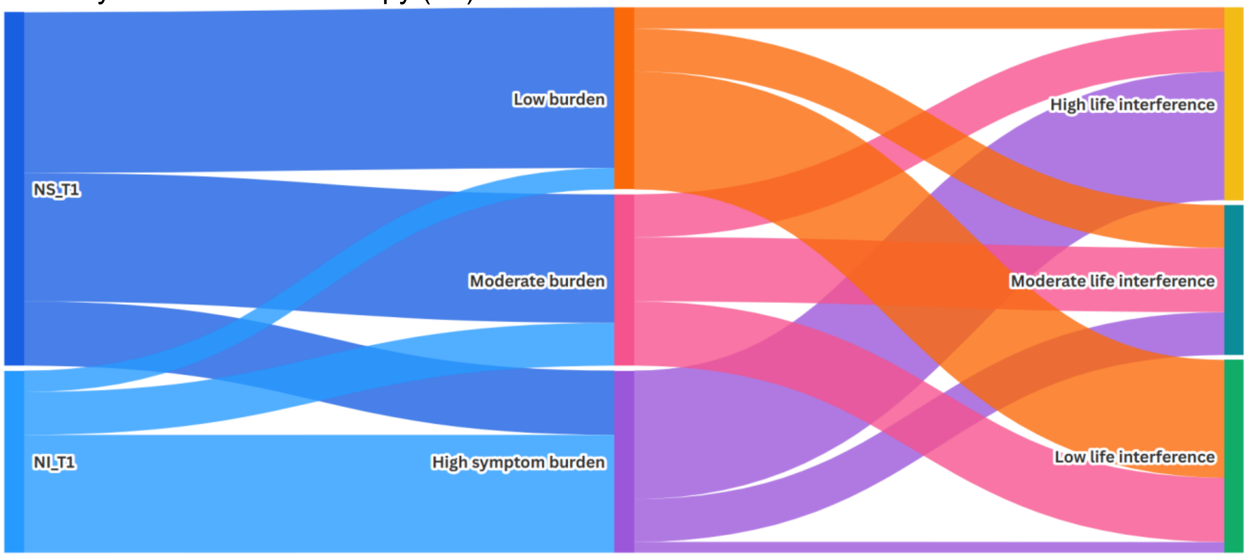

Supplemental Figure S3. Sankey Diagram of NI, Symptom Burden, and Life Interference Pathways in Final Chemotherapy (TFinal) Cohort

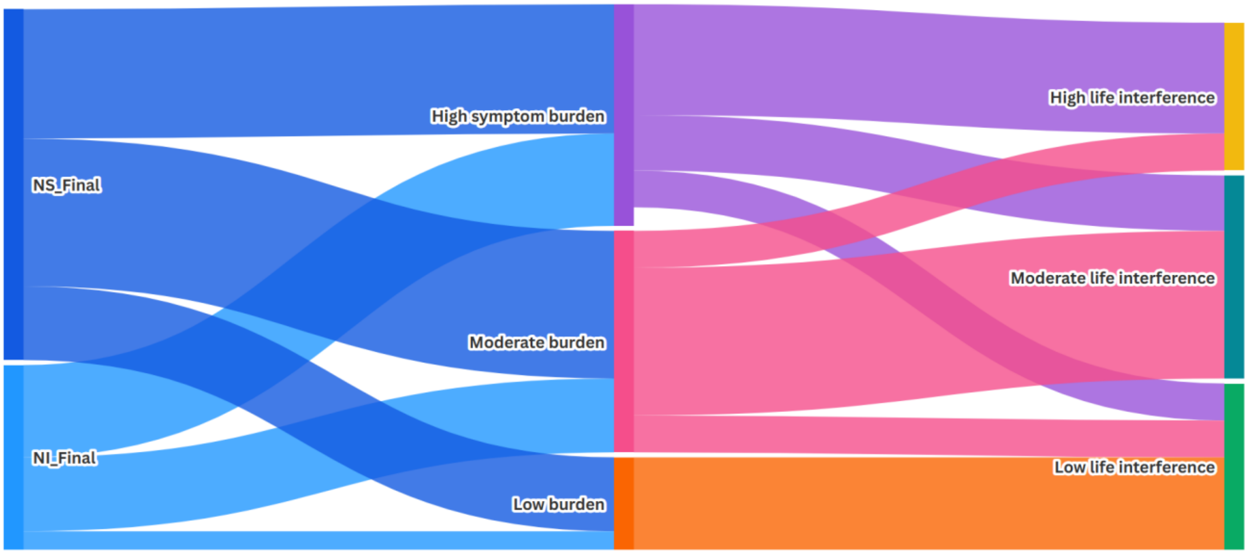

Note for Sankey diagrams: Because T1 and TFinal represent independent cross-sectional samples, these pathways should not be interpreted as longitudinal change.

Supplemental Table S5. Biomarker Differences Between NS and NI Participants in Pre-Chemotherapy (T1) Cohort

Biomarker	NS<br>Mean (95% CI)	NI<br>Mean (95% CI)	F	*p*	Effect Size (η^2)
NPAR	16.0 (CI 14.0-18.1))	14.4 (CI 8.43-20.4)	0.58	0.46	0.02
NLR	3.00 (CI 1.83-4.18)	2.10 (CI 0.70-3.51)	0.86	0.36	0.04
MLR	0.37 (CI 0.27-0.47)	0.32 (CI 0.16-0.48)	0.30	0.59	0.01
PLR	177.4 (CI 122.3-232.5)	128.3 (CI 73.1-183.5)	1.22	0.28	0.05

Supplemental Table S6. Biomarker Differences Between NS and NI Participants in Final Chemotherapy (TFinal) Cohort

Biomarker	NS<br>Mean (95% CI)	NI<br>Mean (95% CI)	F	*p*	Effect Size (η^2)
NPAR	15.2 (CI 12.5-17.9)	15.0 (CI 12.2-17.9)	0.01	0.92	0.00
NLR	4.90 (CI 1.31-8.50)	2.70 (CI 0.87-4.54)	0.78	0.39	0.03
MLR	0.54 (CI 0.37-0.70)	0.62 (CI 0.17-1.08)	0.23	0.64	0.01
PLR	205.8 (CI 79.8-331.8)	141.7 (CI 89.3-194.1)	0.55	0.47	0.02

Supplemental Table S7. Cohort-Level Biomarker Differences Between Pre-Chemotherapy (T1) and Final Chemotherapy (TFinal) Cohorts

Biomarker	Mean (95% CI)	F	*p*	Effect Size (η^2)
NPAR<br>T1<br>TFINAL	 15.6 (13.6-17.6)<br>15.2 (13.2-17.1)	0.11	0.75	0.002
NLR<br>T1<br>TFINAL	 2.75 (1.86-3.65)<br>4.17 (1.76-6.58)	1.21	0.28	0.02
MLR<br>T1<br>TFINAL	 0.36 (0.28-0.44)<br>0.57 (0.40-0.74)	5.04	0.03	0.09
PLR<br>T1<br>TFINAL	 163.7 (122.2-205.1)<br>184.4 (101.1-267.7)	0.20	0.66	0.004

Supplemental Table S8. Spearman Correlations Between Biomarkers and Symptom Burden in Pre-Chemotherapy (T1) and Final Chemotherapy (TFinal) Cohorts

Biomarker	Cohort Timepoint	ρ (Symptom Burden)	*p*
NPAR	T1	-0.12	0.41
	TFinal	0.17	0.36
NLR	T1	-0.16	0.32
	TFinal	0.10	0.57
MLR	T1	0.05	0.78
	TFinal	-0.08	0.64
PLR	T1	0.14	0.38
	TFinal	0.02	0.91

Supplemental Table S9. Spearman Intercorrelations Among Immune-Inflammatory Biomarkers (NPAR, NLR, MLR, PLR)

Biomarker Pair	ρ (Symptom Burden)	*p*
NPAR ↔ NLR	0.53	<0.01
NPAR ↔ MLR	0.58	<0.01
NPAR ↔ PLR	0.72	<0.01
NLR ↔ MLR	0.81	<0.01
NLR ↔ PLR	0.92	<0.01
MLR ↔ PLR	0.67	<0.01
